# Dengue virus infection impedes megakaryopoiesis in MEG-01 cells where the virus envelope protein interacts with the transcription factor TAL-1

**DOI:** 10.1038/s41598-020-76350-5

**Published:** 2020-11-11

**Authors:** Atoshi Banerjee, Aarti Tripathi, Shweta Duggal, Arup Banerjee, Sudhanshu Vrati

**Affiliations:** 1 Translational Health Science and Technology Institute, NCR Biotech Science Cluster, Faridabad, 121001 India; 2 Regional Centre for Biotechnology, NCR Biotech Science Cluster, Faridabad, 121001 India; 3 Present Address: University of Nevada, 4505 S. Maryland Parkway, Las Vegas, NV 89154 USA

**Keywords:** Pathogens, Virology

## Abstract

Dengue virus (DENV) infection causes dengue fever in humans, which can lead to thrombocytopenia showing a marked reduction in platelet counts, and dengue hemorrhagic fever. The virus may cause thrombocytopenia either by destroying the platelets or by interfering with their generation via the process of megakaryopoiesis. MEG-01 is the human megakaryoblastic leukemia cell line that can be differentiated in vitro by phorbol-12-myristate-13-acetate (PMA) treatment to produce platelet-like-particles (PLPs). We have studied DENV infection of MEG-01 cells to understand its effect on megakaryopoiesis and the generation of PLPs. We observed that DENV could infect only naive MEG-01 cells, and differentiated cells were refractory to virus infection/replication. However, DENV-infected MEG-01 cells, when induced for differentiation with PMA, supported an enhanced viral replication. Following the virus infection, the MEG-01 cells showed a marked reduction in the surface expression of platelet markers (CD41, CD42a, and CD61), a decreased polyploidy, and significantly reduced PLP counts. DENV infection caused an enhanced Notch signaling in MEG-01 cells where the virus envelope protein was shown to interact with TAL-1, a host protein important for megakaryopoiesis. These observations provide new insight into the role of DENV in modulating the megakaryopoiesis and platelet production process.

## Introduction

Dengue fever is the most common arthropod-borne viral disease transmitted by the mosquitoes *Aedes aegypti* and *Aedes albopictus*^1^*.* The causative agent of the disease, Dengue virus (DENV), is an enveloped virus with 10.7 kb single-stranded RNA genome encoding three structural proteins; capsid (C), membrane protein (M), and envelope (E); and seven non-structural proteins NS1, NS2a, NS2b, NS3, NS4a, NS4b, and NS5. DENV infection leads to classical dengue fever which is mild and the host immunological response against the virus is capable of subduing it. However, in some cases, dengue fever can progress to thrombocytopenia and a more fatal dengue hemorrhagic fever (DHF) or dengue shock syndrome (DSS). Clinical manifestations of DHF and DSS include continuous high fever for 2–7 days associated with increased vascular permeability leading to plasma leakage, pleural effusions, enhanced hematocrit concentration, and thrombocytopenia, where the platelet count decreases below 15 × 10^4^ per microliter of blood^2–4^. The enhanced platelet destruction and/or suppression of platelet production may be responsible for thrombocytopenia^4,5^.

Megakaryopoiesis, the process of platelet formation from megakaryocyte, is an intricate and dynamic process which depends upon the combinatorial interaction between the cytokine signaling, transcriptional factors and their target genes^6,7^. Within the bone marrow, hematopoietic stem cells undergo lineage-specific commitment to form megakaryocytes which further undergo maturation, endoreplication leading to polyploidy, and formation of pro-platelet processes^6^. From the pro-platelet processes, platelets are shed into the vascular sinusoids within the bone marrow. Thus, any disruption of the megakaryopoiesis process can lead to thrombocytopenia. There is limited information on the effect of DENV infection on megakaryopoiesis. Basu et al. showed that DENV inhibits in vitro megakaryocytic colony formation from CD34+ human cord blood cells^8^. Recently, Lin et al. demonstrated the suppressive effect of domain III of DENV E protein on megakaryopoiesis in human cord blood-derived CD34+ cells and mouse bone marrow cells^9^.

The human megakaryoblastic leukemia cell line MEG-01 shows phenotypic properties resembling closely to those of megakaryoblasts^10^. DENV (serotype 2) replicated efficiently in undifferentiated MEG-01 cells^11^. These cells have been shown to differentiate in the presence of phorbol-12-myristate-13-acetate (PMA) and release platelet-like particles (PLPs)^12^. We have established an in vitro model of platelet production from MEG-01 cells and show that DENV infection alters the megakaryopoiesis in these cells leading to the lowering of platelet numbers.

## Methods

### Virus and cells

Dengue virus serotype 2 (DENV-2) (IND/P23085/1960 strain, Gene Bank accession no. JQ922552.1) was used in this study. The virus was cultured in C6/36 cells and concentrated through centricon-100 K before aliquoting and storing at − 80 °C. The virus was titrated by focus-forming unit (FFU) assay on Vero cells using D1-4G2-4-15 antibody^13^. The *Aedes albopictus* C6/36 cells (CRL-1660, ATCC) were maintained in the L-15 medium at 28 °C The African green monkey kidney Vero cells (National Centre for Cell Science, Pune, India) were maintained in Minimal Essential Medium (MEM) (Invitrogen), and the human megakaryoblast MEG-01 cells (CRL-2021, ATCC) were maintained in RPMI 1640 medium at 37 °C with 5% CO_2_. All culture media were supplemented with 10% Fetal Bovine Serum (FBS, HyClone) and 1% Penicillin–Streptomycin–Glutamine.

### Antibodies

Antibodies used were as follows: Notch-1 (4380T, Cell Signaling), DLL-1 (ab85346, Abcam), RBPjk (ab180588, Abcam), TAL-1 (PA5-30586, Invitrogen), GAPDH (GTX100118, GeneTex), DENV envelope monoclonal antibody D1-4G2-4-15 (HB-112, ATCC). HRP-conjugated secondary antibodies were purchased from Jackson Immunochemicals. Fluorescence-conjugated Alexa fluor 488 and 647, and Annexin V-alexa 488 were from Invitrogen. FACS antibodies CD61-APC and CD42a-Alexa 488 were from BD Biosciences and CD41-PE from Biolegend.

### Quantitative real-time PCR (qRT-PCR)

The total RNA was isolated from cells using Trizol reagent (Thermofisher) according to the manufacturer's protocol. For cDNA synthesis, 1 µg RNA was used with random hexamers, dNTPs, and reverse transcriptase (PrimeScript™ 1st strand cDNA Synthesis Kit, Takara). Specific primers were used along with SYBR green mix to perform the qRT-PCR (SYBR® Premix Ex Taq™ II, Tli RNase H Plus, Takara) in a Real-time thermocycler (Applied Biosystems 7500). The relative expression was normalized to *Gapdh* expression. The sequence of the primers used were: GARAGACCAGAGATCCTGCTGTCT (DENV forward primer), ACCATT CCATTTTCTGGCGTT (DENV reverse primer), CGTCCCGTAGACAAAATGGT (*Gapdh* forward primer), TTGATGGCAACAATCTCCAC (*Gapdh* reverse primer), GCAGTTGTGCTCCTGAAGAA (*Notch-1* forward primer), CGGGCGGCCAGAAAC (*Notch-1* reverse primer).

### Generation of platelet-like-particles (PLPs)

MEG-01 cells were stimulated with 50 ng/ml PMA and incubated at 37 °C for 5 days. Culture supernatant was collected and centrifuged at 150 g for 10 min to remove cells, followed by 500 g for 10 min for debris removal. The supernatant was then centrifuged at 1000 g for 15 min to pellet the PLPs. The phosphate-buffered saline (PBS)-washed PLPs were stained with CD41 antibody for 1 h on ice and identified by size and shape (side and forward scatter) where the normal human platelets would fall, and the presence of CD41 marker. Ten thousand events were recorded and the PLP count was quantified by the rate (events/s)^14^.

### Surface marker analysis

For the surface marker analysis, cells and PLPs were harvested separately, washed with PBS, and stained with CD61-APC, CD42a-Alexa 488, and CD41-PE antibodies for 1 h on ice in dark. Cells and PLPs were determined based on SSC and FSC in FACSCanto (BD Biosciences). For multicolor analysis, compensation was carried out using unstained and single-color samples, and compensated voltages were applied to all experimental samples. All the samples were acquired in the same compensated setting and analysis was done by FlowJo software (Version 10.6.2).

### Annexin V labeling

For Annexin V labeling, cells were washed with PBS and resuspended in Annexin V Binding Buffer (BD Biosciences). One µl of Annexin V-alexa 488 was added to 100 µl of cell suspension (1 × 10^6^ cells) and kept in dark for 15 min on ice. Cells were immediately acquired by FACSCanto (BD Biosciences). Annexin V positive and negative cells were gated and analyzed.

### Polyploidy analysis

For polyploidy analysis, cells were harvested, washed with PBS, fixed with 70% ethanol and kept at − 20 °C till use. On the day of analysis, cells were washed twice with PBS and treated with 100 µg/ml RNAse A and 40 µg/ml propidium iodide (PI) for 30 min in dark at 37 °C. Cells were acquired in FACSCanto (BD Biosciences) and stages of cell cycle 2 N, 4 N, and > 4 N population were analyzed by FlowJo software.

### May–Grünwald–Giemsa stain

For May–Grünwald–Giemsa staining, cells were fixed with ice-cold methanol for 5 min and washed with water. May–Grünwald stain (1:1 in water) was added to cells for 5 min and changed with Giemsa stain (1:9 in water) for 30 min without washing. Cells were washed with distilled water thrice and observed under a microscope.

### Preparation of nuclear and cytoplasmic fractions

MEG-01 cells were harvested by centrifugation and the nuclear and cytoplasmic extracts were prepared using the NE-PER Nuclear and Cytoplasmic Extraction Reagents (Thermo Scientific) as per the recommended protocol by the manufacturer.

### Co-immunoprecipitation

The endogenous Tal-1 pull-down was performed at 4 °C by overnight incubation of protein lysate with rabbit anti-Tal-1 antibody (Invitrogen, PA5-30586) or rabbit IgG (Cell Signaling, 2729S) as the isotype control, followed by 3 h incubation with Protein A/G magnetic beads (Pierce, 88802) at 4 °C. The antibody-protein complex was pulled down and separated on a 10% SDS-PAGE, followed by Western blotting with DENV-2 anti-envelope monoclonal antibody D1-4G2-4-15 (ATCC).

### Confocal microscopy

Cells were PMA-stimulated on coverslips, washed with PBS and fixed with 2% paraformaldehyde for 15 min, followed by permeabilization with 0.03% Triton-X 100 in PBS for 1 h. Blocking was done in 2% bovine serum albumin (BSA) in PBS for 1 h, primary antibody addition for 1 h was followed by fluorescent-labeled secondary antibody addition for 1 h in dark. Cells were mounted on slides using ProLong Gold anti-fade reagent with DAPI. Images were taken at 100X using an Olympus FV1000 confocal microscope. For the colocalization studies, Z-stacks were acquired at 0.25 µm per slice in sequential scanning. Images were analyzed by Fluoview software (version 4.2) and Pearson's coefficient calculated at different regions of interest (ROI). Pearson's values are a mean of 10–15 ROIs observed in different cells.

### Statistical analyses

Statistical analyses were done using the one-way analysis of variance (ANOVA) and a *p* value ≤ 0.05 was considered significant. Sigma plot (version 10) was used for graphs and analysis.

## Results

### PMA treatment of MEG-01 cells induces differentiation and production of platelet-like particles

**Figure 1:**
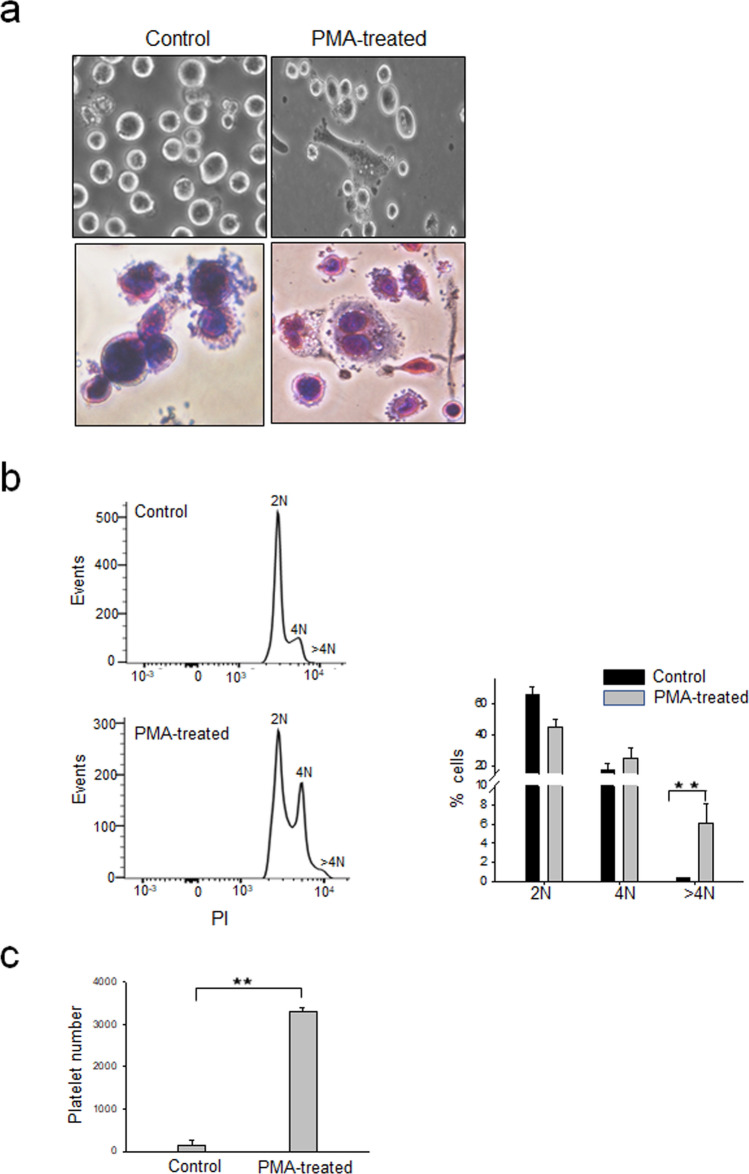
PMA treatment of MEG-01 cells induces differentiation and enhanced production of platelet-like particles. (**a**) MEG-01 cells were incubated for 3 days in RPMI 1640 medium (control) or in RPMI 1640 containing 50 ng/ml PMA (PMA-treated) and photographed at 20× resolution under an inverted light microscope (upper panels). Untreated control cells or PMA-treated MEG-01 cells were fixed with Methanol and subjected to May-Grünwald-Giemsa staining and visualized at 20× resolution under an inverted light microscope (lower panels). Experiment were repeated 3 times and representative images are shown. (**b**) MEG-01 cells treated as above were fixed and stained with PI and acquired by FACS for analyzing the cell cycle. The 2 N, 4 N, and > 4 N population were analyzed by FlowJo software. The left panel shows data from a typical experiment. The data in the right panel is from 3 replicates of the experiment. (**c**) MEG-01 cells were incubated in RPMI 1640 with or without PMA (50 ng/ml) for 5 days. The culture supernatant was collected and processed for PLP preparation. The PLPs were stained with CD41 antibody and analyzed by FACS. PLPs were identified by size and shape (side and forward scatter) where normal human platelets would fall and the presence of CD41 marker. Ten thousand events were recorded and platelet count was quantified by the rate (platelet events/min). The data is from 3 independent experiments.

PMA treatment of MEG-01 cells is known to induce differentiation leading to polyploidy and production of platelet-like particles (PLPs)^15,16^. In the present study, MEG-01 cells, when treated with PMA, differentiated to form cytoplasmic extensions and showed elongated morphology. Staining of these cells with May–Grünwald–Giemsa stain showed an increase in cell size with multiple nuclei. Cells with protrusions and extended cytoplasmic processes resembling megakaryocytes displaying proplatelets were clearly visible (Fig. 1a). Following the treatment of MEG-01 cells with PMA, the number of 4 N cells and > 4 N cells increased (Fig. 1b) with a significantly enhanced production of PLPs (Fig. 1c). The PMA induction of MEG-01 cells thus mimics the megakaryopoiesis process in vitro in a cell culture model.

### DENV productively infects MEG-01 cells

**Figure 2:**
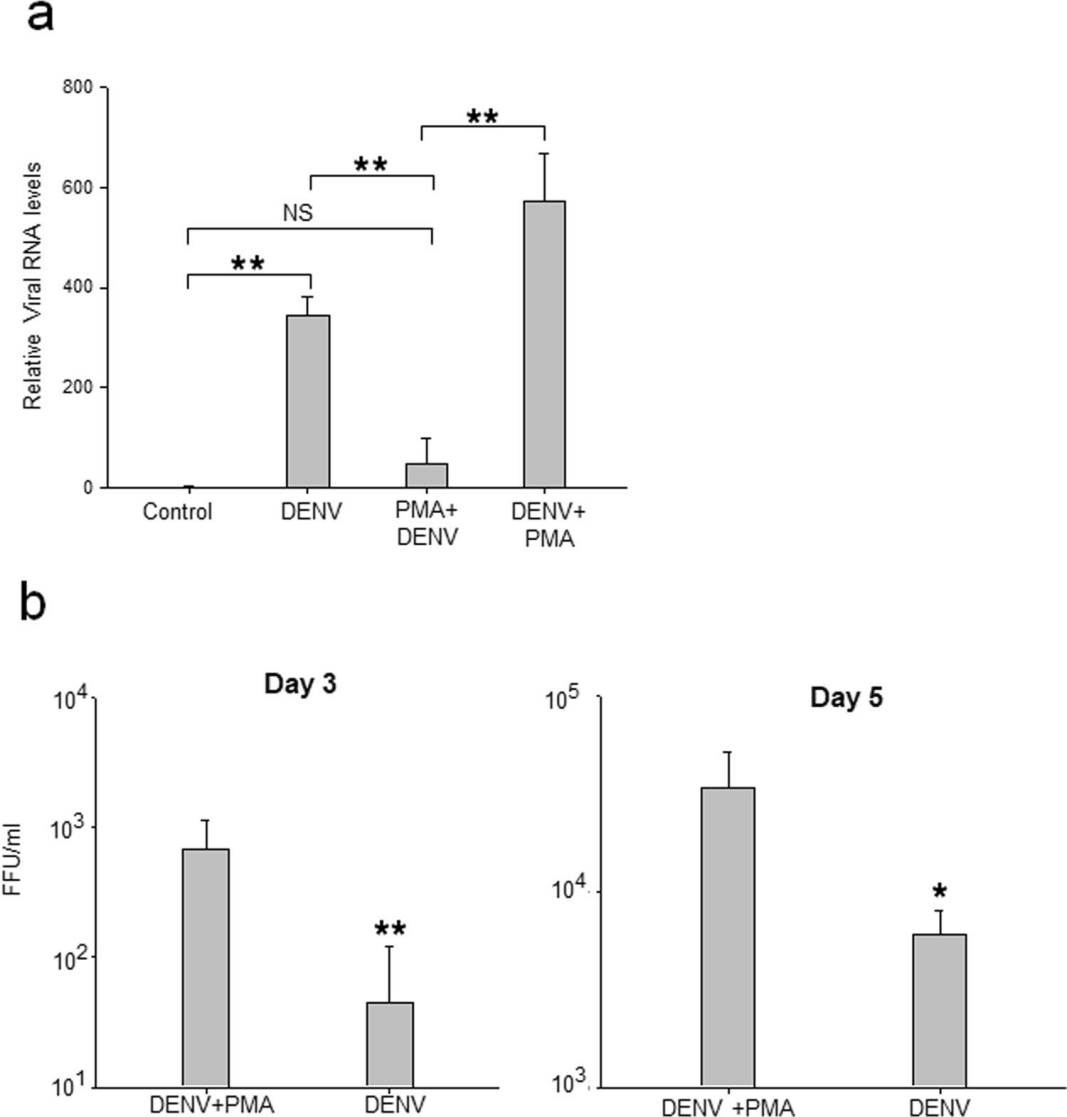
DENV infection of MEG-01 cells. MEG-01 cells were mock-infected, or infected with DENV at a multiplicity of infection (MOI) 1, and the cells and culture supernatant were harvested on different days post-infection (pi). In parallel, cells were pre-treated with PMA 2 h before infection (PMA + DENV) or infected with DENV followed by PMA treatment (DENV + PMA). (**a**) The total RNA from cells was isolated on day 2 pi and relative levels of viral RNA determined by qRT-PCR using *Gapdh* transcripts for normalization. (**b**) The culture supernatants from virus-infected cells were collected on days 3 and 5 pi and DENV titers determined by FFU assays. All the experiments were done more than 3 times and data represent the mean ± standard deviation. Statistical analyses were done using the one-way analysis of variance (ANOVA). *p < 0.05 and **p < 0.005.

MEG-01 cells were incubated with DENV, and the susceptibility of cells to virus infection was studied by determining the levels of viral genomic RNA in cells on day 2 post-infection (pi). To examine if PMA treatment of MEG-01 cells (and the initiation of differentiation process) had any effect on cell susceptibility to DENV infection, cells were incubated with PMA 2 h before (PMA pre-treatment) or 2 h after DENV infection (post-infection), and levels of viral RNA determined on day 2 pi. While the untreated (and therefore undifferentiated) MEG-01 cells were readily infected with DENV, the PMA pre-treated cells showed a significantly lower virus replication (Fig. 2a). However, post DENV infection PMA-treatment did not lower DENV replication. In fact, a significantly higher viral RNA replication was seen in these cells (Fig. 2a). To corroborate this, DENV titers in the culture supernatant on day 3 and day 5 pi were determined. As can be seen in Fig. 2b, DENV replication was significantly higher in post infection PMA-treated cells. On day 5 pi, around 50% cells showed DENV infection. No difference was apparent in viability of DEN + PMA cells as compared to Mock + PMA cells. No DENV could be detected by FFU assay in PMA-pretreated DENV-infected cell culture supernatant.

These data show that naïve MEG-01 cells were susceptible to DENV infection while PMA-pretreated, differentiated MEG-01 cells were refractory to virus infection/replication. Additionally, once infected with DENV, post-infection PMA treatment lead to differentiation of MEG-01 cells and higher DENV replication. Here onwards, PMA treatment was given post DENV infection and referred as DENV + PMA.

### Dengue virus infection of MEG-01 cells reduces polyploidy and production of PLPs

**Figure 3:**
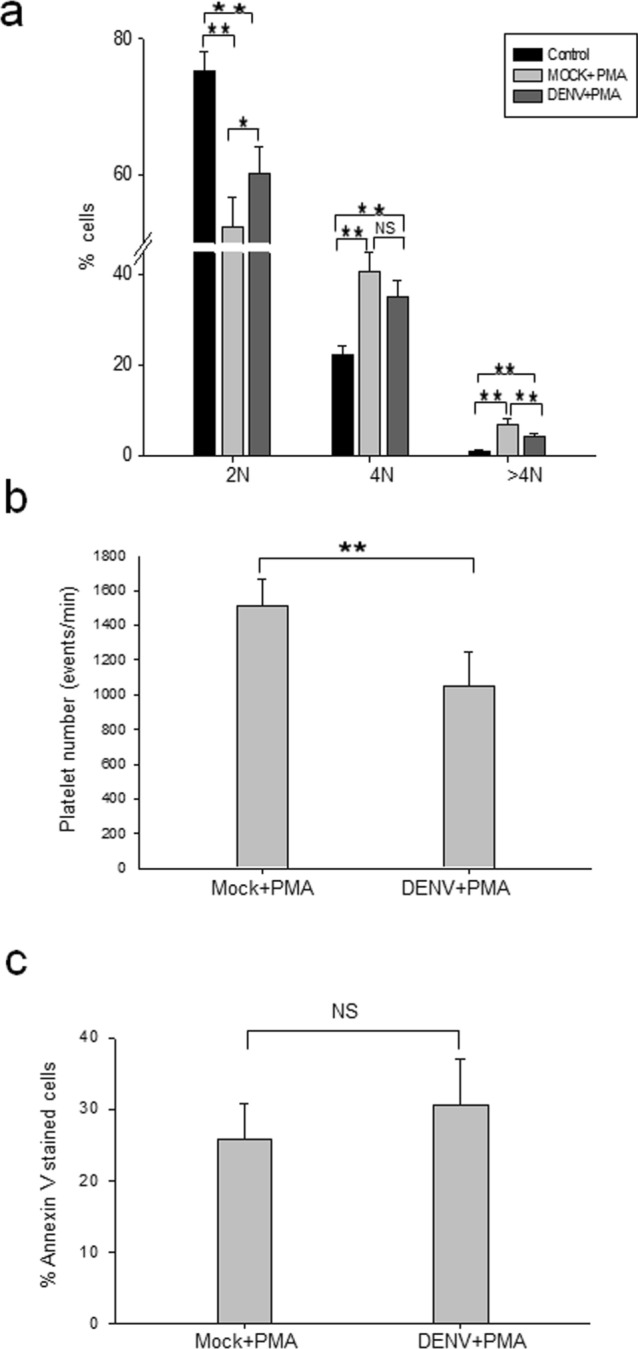
DENV infection of MEG-01 cells reduces polyploidy and production of PLPs. (**a**) MEG-01 cells were infected with DENV (MOI 1). DENV + PMA and Mock + PMA cells, along with naïve MEG-01 cells (untreated control) were incubated in culture medium at 37 °C for 5 days. Cells were fixed and stained with PI, and acquired by FACS for analyzing the cell cycle. The 2 N, 4 N, and > 4 N populations were analyzed by FlowJo software. (**b**) Culture supernatant from Mock + PMA and DENV + PMA treated cells were collected and processed for isolation of PLPs. The PLPs were stained with CD41 antibody and analyzed by FACS. PLPs were identified by size and shape (side and forward scatter) where the normal human platelets would fall and the presence of CD41 marker. Ten thousand events were recorded and platelet count was quantified by the rate (platelet events/min). (**c**) Cells harvested at day 5 pi were stained with Annexin V-alexa 488 antibody and acquired by FACS. Annexin V positive and negative cells were gated and analyzed. All the experiments were done more than 6 times and data represent the mean ± standard deviation. Statistical analyses were done using the one-way analysis of variance (ANOVA). *p < 0.05 and **p < 0.005.

PMA-induced megakaryopoiesis in MEG-01 cells involves the development of polyploidy that is followed by the generation of PLPs^15,16^. To study if PMA-induced polyploidy in MEG-01 cells was affected by DENV infection, the nuclei in the cells were visualized by PI staining of DNA. As expected, PMA treatment of MEG-01 cells significantly enhanced the number of 4 N and > 4 N cells (Fig. 3a). Compared to the mock-infected MEG-01 cells treated with PMA, the number of > 4 N cells was significantly reduced in DENV + PMA treated cells. In the experiment reported here, there was 36% reduction in the number of > 4 N cells in the DENV + PMA cells (*p* < 0.005). Although the numbers varied from experiment to experiment, the reduction seen here was reproducible.

The data above showed that DENV infection affected the process of polyploidy which may have implications for the generation of platelets. We counted the number of PLPs based on their size, shape and CD41 gating by FACS and found that it was ~ 28% lower in DENV + PMA cells than that seen in the mock-infected, PMA-treated cells (Mock + PMA), and this difference was statistically significant (Fig. 3b).

Apoptosis is required for the platelet biogenesis in general^17–19^. During the MEG-01 cell differentiation following PMA stimulation, some intrinsic apoptosis pathways may be initiated resulting in a reduction in the number of the polyploid cells and the PLPs seen above. This was studied by Annexin V staining. Annexin staining was recorded in ~ 25% Mock + PMA cells and ~ 30% DENV + PMA cells (Fig. 3c), and this difference was statistically not significant. Thus, reduction in polyploidy and PLP numbers seen in DENV-infected MEG-01 cells may not be due to apoptosis and could be related to other biochemical consequences of virus infection.

### Dengue virus infection arrests surface marker expression acquired during MEG-01 cell differentiation

**Figure 4:**
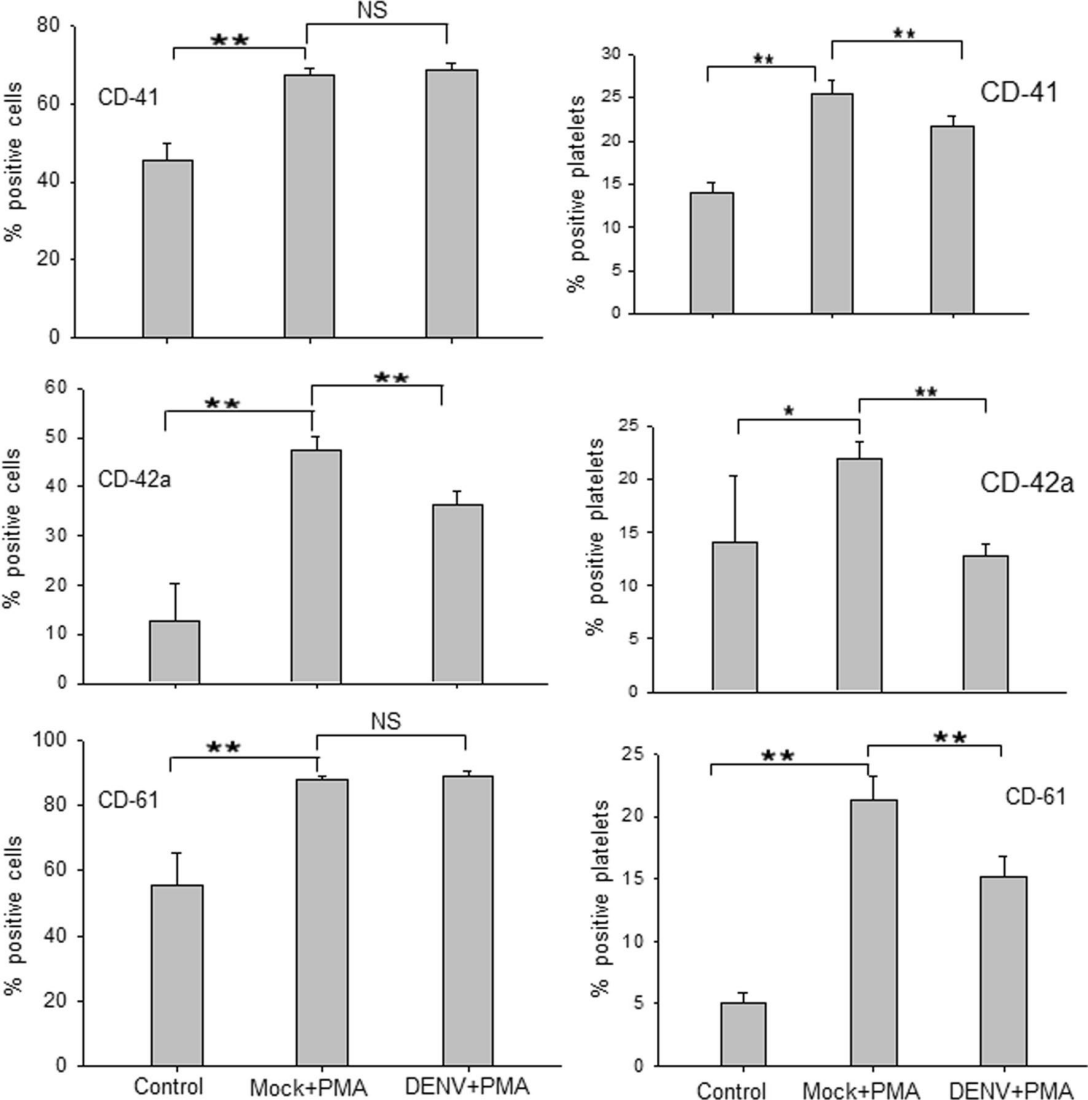
DENV infection arrests surface marker expression acquired during MEG-01 cell differentiation. Naïve MEG-01 cells (control) were treated with PMA (Mock + PMA) or DENV-infected (MOI-1) cells were treated with PMA (DENV + PMA). Cells and PLPs were harvested at day 5 pi and stained with CD61-APC, CD42a-Alexa 488, and CD41-PE antibodies. Cells and platelets were determined based on SSC and FSC by FACS. For multicolor analysis, compensation was carried out using unstained and single-color samples and compensated voltages were applied to all the experimental samples. All the samples were acquired in the same compensated setting and analysis was done by FlowJo software. All the experiments were done more than 6 times and data represent the mean ± standard deviation. Statistical analyses were done using the one-way analysis of variance (ANOVA). *p < 0.05 and **p < 0.005, NS denotes difference not-significant.

During the PLP formation, morphological and functional changes are observed in MEG-01 cells with the expression of distinct surface markers, characterizing different stages of megakaryopoiesis^16^. To examine if DENV infection induced any changes in the levels of megakaryopoiesis-specific cell surface markers on MEG-01 cells, we collected the virus-infected cells and PLPs on day 5 pi, followed by staining with CD41, CD42a and CD61 antibodies for FACS analysis. As anticipated, PMA-treated MEG-01 cells showed significantly higher surface expression of CD41, CD42a and CD61 markers compared to those seen on control cells (Fig. 4). In DENV + PMA cells, we observed that CD41 and CD61 expression was similar to that in Mock + PMA, however; CD42a levels were ~ 11% lower in DENV + PMA treated cells.

It may be noted that different markers affect platelet biogenesis differently, like Bernard-Soulier syndrome has decreased CD42a and Glanzmann thrombasthenia has decreased CD61^20,21^. The differences seen above in the three markers may be related to the stage of cell differentiation and a hallmark of megakaryopoiesis process.

As expected, the levels of CD41, CD42a, and CD61 were higher in PLPs produced from PMA-treated MEG-01 cells when compared with untreated control cells (Fig. 4). Importantly, levels of these markers were significantly lower in PLPs produced from DENV + PMA cells in comparison to those produced from Mock + PMA cells. This may indicate the relatively immature nature of platelets produced from DENV-infected cells that may result in the compromised ability of these platelets to maintain homeostasis during thrombosis.

### Dengue virus infection induces Notch signaling in MEG-01 cells

The process of megakaryopoiesis and platelet production is complex and achieved by tight regulation between various signaling pathways. Thrombopoietin (TPO) is the major regulator of megakaryopoiesis along with other pathways like MAPK, JAK/STAT, ERK and Notch^22^. The role of Notch signaling in the process of megakaryopoiesis has been studied in great detail^23–27^. The Notch pathway enhances megakaryopoiesis in the mouse system, whereas in human cells the reverse is true. Nevertheless, these studies clearly demonstrate that Notch signaling has a crucial role in megakaryopoiesis in both human and murine systems.

**Figure 5:**
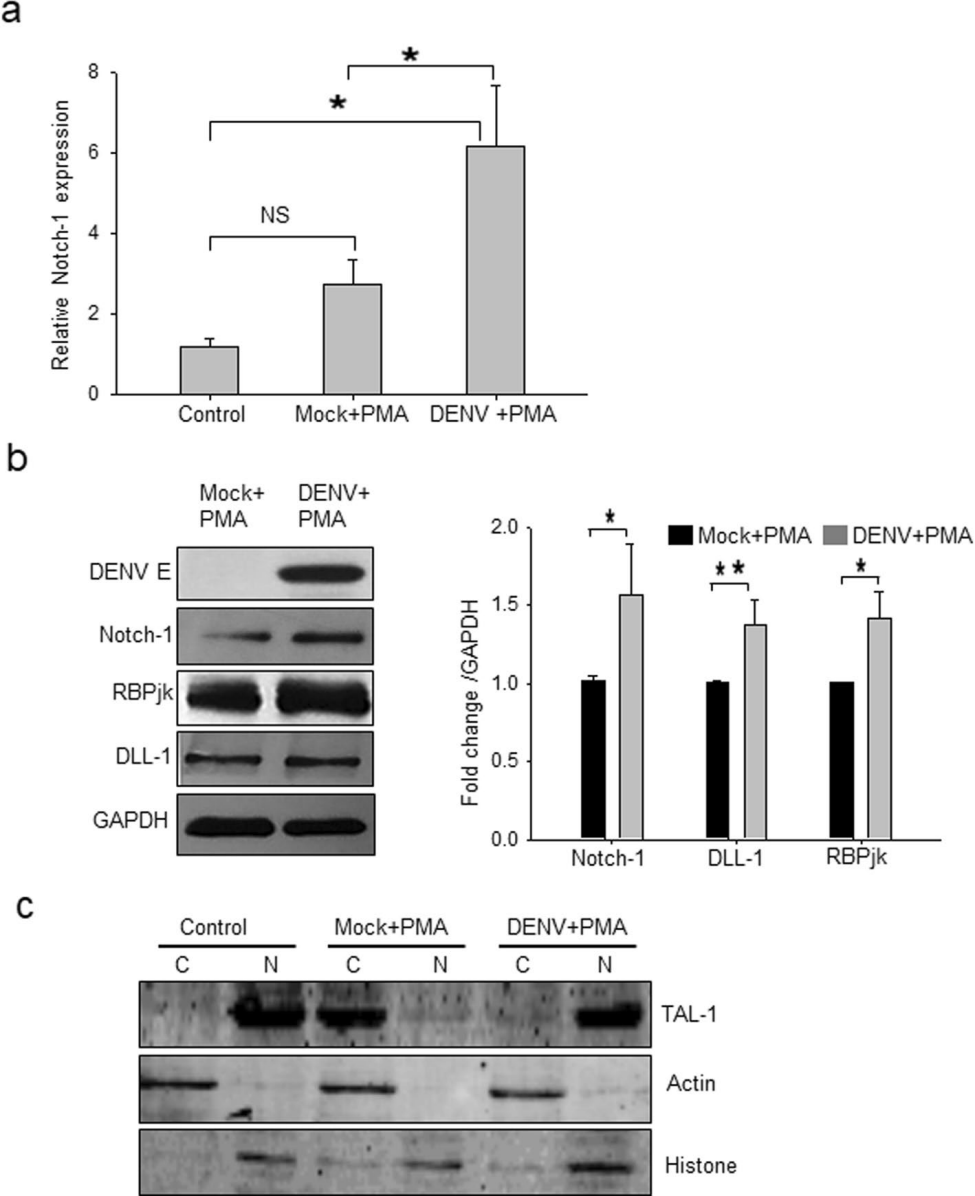
Notch signaling in DENV-infected MEG-01 cells. Naïve MEG-01 cells (control) were treated with PMA (Mock + PMA), or DENV-infected (MOI-1) cells were treated with PMA (DENV + PMA). Cells were harvested at day 5 pi for isolating total RNA and preparation of cell lysate. (**a**) Expression of *Notch-1* transcript relative to *Gapdh* expression is presented from 3 independent experiments. (**b**) The cell lysate was Western blotted for different proteins (left panel) and the band intensities were quantified by ImageJ software. Intensities from 3 or more blots were determined compared to GAPDH, and average intensities in DENV + PMA compared to Mock + PMA treatment were plotted to show the fold-change in protein expression (right panel). Intensities from 3 or more blots were determined. (**c**) Nuclear (N) and cytoplasmic (C) extracts of MEG-01 cells were prepared and Western blotted with TAL-1 antibody. Levels of Actin and Histone proteins were checked to demonstrate the quality of protein fractionation. Statistical analyses were done using the one-way analysis of variance (ANOVA). *p < 0.05, **p < 0.005, NS denotes difference not-significant.

DENV infection is known to upregulate Notch ligands like DLL-1 and DLL-4 in monocytes^28^. We, therefore, studied if DENV infection affected Notch signaling in MEG-01 cells. We observed that PMA treatment of cells upregulated Notch-1 receptor transcript expression 2.7-folds compared to untreated control cells, although this was not statistically significant (Fig. 5a). Interestingly, DENV + PMA cells showed 6.2-folds higher expression (*p* < 0.05) of Notch-1 transcript compared to the untreated control cells and ~ 2-folds higher expression (*p* < 0.05) compared to that in Mock + PMA cells, suggesting that DENV infection might play a role in enhancing Notch-1 receptor levels on MEG-01 cells. Expression of other Notch receptors, namely Notch-2, Notch-3, and Notch-4, was undetectable at transcript level.

We next examined whether DENV infection modulated the levels of Notch-1 and its signaling components at the protein level by Western blotting. Notch-1 protein levels were 1.6-folds higher in DENV + PMA cells, compared to that in the Mock + PMA cells. The levels of DLL-1 and RBPjk, the downstream effectors of Notch pathway, were also higher by ~ 1.4-folds in virus-infected cells (Fig. 5b).

It is known that Notch-1 and megakaryopoiesis-specific transcription factor TAL-1 are inter-dependent^29,30^. Western blotting was done to examine the TAL-1 status in DENV-infected MEG-01 cells. TAL-1 was found exclusively in the nucleus in untreated MEG-01 cells whereas in Mock + PMA cells it was found mostly in the cytoplasm. In DENV + PMA cells, TAL-1 was found mostly in the nucleus but a small amount of it was seen in the cytoplasm as well (Fig. 5c).

### Interaction of TAL-1 and DENV E protein in virus-infected MEG-01 cells

**Figure 6:**
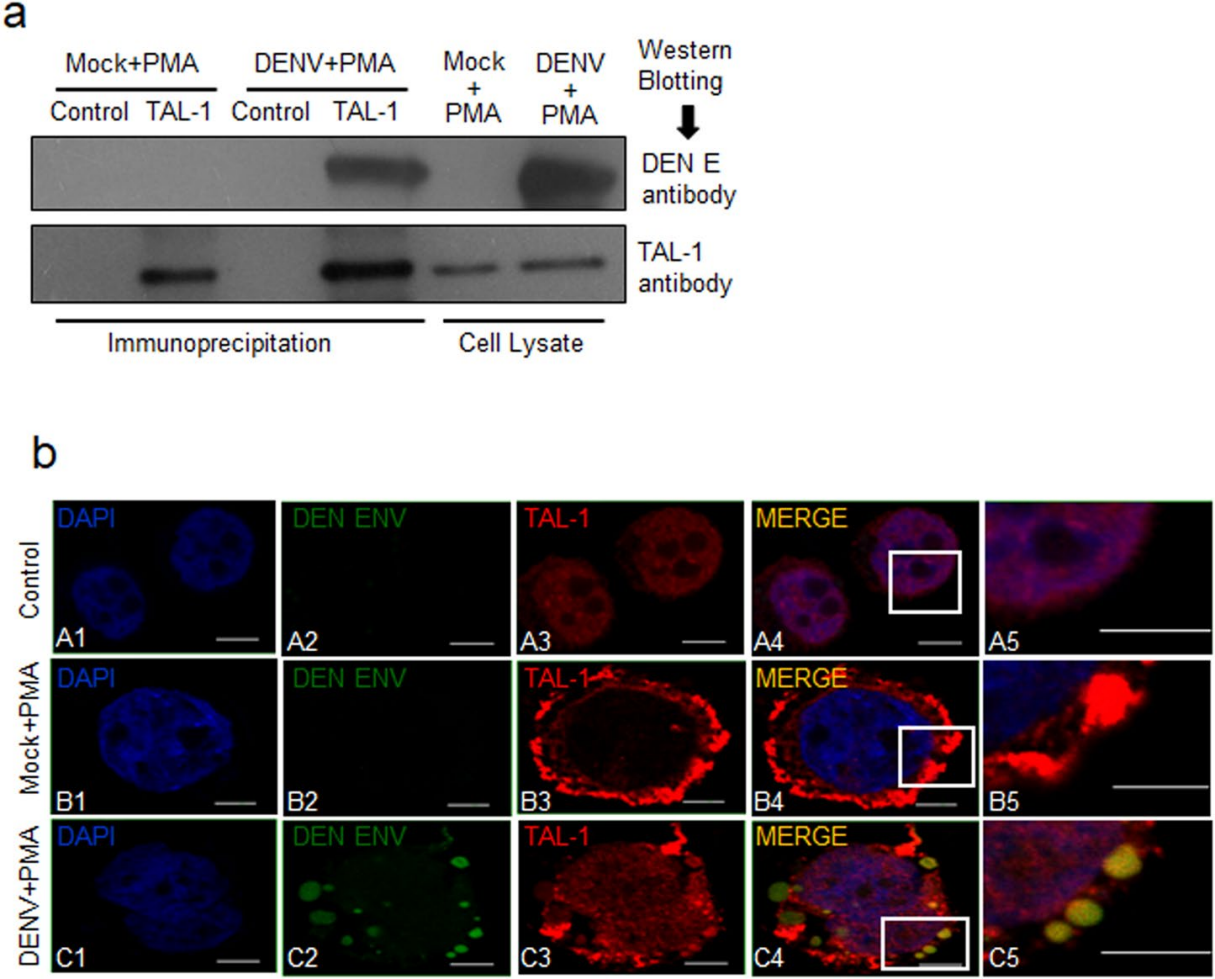
Interaction of Tal-1 and DENV E protein. Naïve MEG-01 cells (control) were treated with PMA (Mock + PMA), or DENV-infected (MOI-1) cells were treated with PMA (DENV + PMA). (**a**) Cells were harvested at day 5 pi for preparation of cell lysate. Immunoprecipitation was carried out using the rabbit anti-TAL-1 antibody, or the rabbit IgG as the isotype control, and the Protein A/G magnetic beads. The precipitated proteins and the cell lysate used for the immunoprecipitation were Western blotted with antibodies shown on the right side of the figure. (**b**) On day 5 pi, cells were harvested, fixed in 2% paraformaldehyde, followed by permeabilization with 0.03% Triton-X. This was followed by incubation with TAL-1, and DENV E antibodies and stained with the fluorescent-labeled secondary antibody. Cells were mounted on slides using ProLong Gold anti-fade reagent with DAPI and images taken using a confocal microscope. Untreated MEG-01 cells were used as a control. Experiments were performed 3 times and representative confocal images are shown. The right-most panels are zoomed up images of the insets in the adjacent panels. The scale bar size is 5 μm.

Since transcription factor TAL-1 plays an important role in megakaryopoiesis, and that DENV infection was found to interfere with this process in MEG-01 cells, we sought to examine if there was an interaction of DENV protein/s with TAL-1 in virus-infected MEG-01 cells. To study this, immunoprecipitation was performed with TAL-1 specific antibody on cell lysate prepared from DENV + PMA or Mock + PMA cells. Interestingly, TAL-1 co-immunoprecipitated DENV E protein (Fig. 6a), suggesting an interaction between these two proteins in DENV + PMA cells. The anti-E antibody was not suitable for immunoprecipitation and so reverse co-immunoprecipitation could not be undertaken.

Localization of TAL-1 with DENV E protein in virus-infected MEG-01 cells was further studied by confocal microscopy (Fig. 6b). We found that TAL-1 localized to the nucleus in untreated cells (panel A3). However, TAL-1 localized mostly to the cytoplasm of Mock + PMA cells and distributed throughout (panel B3) whereas, in DENV + PMA cells, TAL-1 protein could be seen in the cytoplasm as well as the nucleus (panel C3). DENV infection showed distinct punctate formation in MEG-01 cells where DENV E protein was localized (panel C2). Almost 90% of virus-infected cells showed punctate phenotype. Such punctate structures have been reported in cells during the DENV replication^31^. When TAL-1 and DENV E signals were merged, we found that TAL-1 nicely colocalized with DENV E protein (Pearson’s coefficient 0.67) in the cytoplasm (panels C4 and C5). These data showed that TAL-1 interacted with DENV E protein in DENV-infected MEG-01 cells.

## Discussion

In this study, we have provided evidence that DENV infection could affect the formation of platelets by hindering the process of megakaryopoiesis. We observed the induction of the Notch pathway in MEG-01 cells during the DENV infection. Notch signaling has been known to suppress megakaryopoiesis in human cells^24,26^. Using the co-immunoprecipitation and colocalization experiments, we demonstrated that DENV E protein interacted with megakaryopoiesis-specific transcription factor TAL-1 in MEG-01 cells. Together these findings present a plausible mechanism of DENV-induced thrombocytopenia and establish the utility of MEG-01 cells as an in vitro model for studying megakaryopoiesis and platelet biology during dengue infection.

Consistent with the previous report of DENV infection of bone marrow cells in a non-human primate model of disease^32^, our study conclusively established that DENV could infect undifferentiated MEG-01 cells, and the cell differentiation, that followed the virus infection, supported an enhanced virus replication. Higher levels of viral replication in differentiated cells advocated that the virus used the altered cellular machinery of the differentiated cells more efficiently for its replication. Since the differentiated MEG-01 cells were refractory to DENV infection, it can be speculated that PMA-pretreatment of cells, leading to differentiation, resulted in an altered protein expression affecting a key molecule(s) with a role in cell surface receptor for DENV entry or a downstream step in virus replication. However, this was not investigated further.

Our data demonstrated that DENV infection of MEG-01 impeded the process of megakaryopoiesis. Our data also showed that DENV infection upregulated the Notch signaling pathway in these cells. It is well established that Notch signaling in human system inhibited megakaryopoiesis and platelet formation^24,26^. The DENV-induced Notch signaling may thus have a role in impeding megakaryopoiesis and generation of platelets in human patients. It is known that Notch-1 not only regulates its downstream factor RBPjk^33^ but also regulates megakaryopoiesis-specific transcription factor TAL-1^29^. It can be argued that sequestering of TAL-1 by DENV E protein in the cytoplasm could affect its availability for its transcription related function in the nucleus, thereby hindering the megakaryopoiesis, and thus the platelet formation. It is possible that pathways other than the Notch pathway are also affected during DENV infection that could affect megakaryopoiesis, and this needs to be studied further. DENV serotype 2 was used in the current study. It would be interesting to explore if other DENV serotypes affect megakaryopoiesis as well by interacting with TAL-1.

Here we show that the decreased platelet formation in DENV-infected MEG-01 cells is not due to apoptosis. Chu et al. have shown that DENV infection causes autophagosome formation^34^. DENV-induced autophagy, thus, could potentially contribute to the decreased platelet formation in DENV-infected MEG-01 cells; and this may be studied in future.

In our study, level of several platelets surface-specific markers (CD41, CD42a, and CD61) declined in the DENV-infected MEG-01 cells. These observations suggest that platelets produced from DENV-infected cells might have a compromised platelet aggregation ability. Similar to Glanzmann thrombasthenia, DENV-infected platelets showed a low level of CD41-CD61 complex^21^ that may result in inefficient platelet binding to fibrinogen and increased possibility of bleeding^35^. Additionally, decreased CD41 on platelets as observed in the present study, is the hallmark of Bernard–Soulier syndrome associated with thrombocytopenia^20,36^. Altogether, a reduction in the levels of important surface markers on platelets seen here in DENV-infected cells may result in the loss of adhesion and thrombocytopenia.

An important step in megakaryopoiesis is endomitosis, where mitosis is aborted mid-anaphase and the cell reenters mitosis^37^. Thus, the chromosome number increases within a single lobulated nucleus resulting in polyploidy. Polyploidy is one of the paramount processes which allow amplification of genome to support extensive protein synthesis necessary for cell growth^38^, a characteristic of megakaryocyte development, and eventually platelet formation. It is evident from our study that DENV infection suppresses the polyploidy process and thus affecting platelet formation.

In conclusion, we show that DENV E protein binds TAL-1, a host factor important for megakaryopoiesis, in DENV-infected MEG-01 cells. This could potentially affect the availability of free TAL-1 and impede the process of megakaryopoiesis either directly or in combination with other factors, resulting in the decreased platelet numbers seen in the dengue patients.

## Supplementary information

Supplementary Information.
