# Supplementary figures and images for: Dengue virus infection impedes megakaryopoiesis in MEG-01 cells where the virus envelope protein interacts with the transcription factor TAL-1
Source: Sci Rep. 2020 Nov 11;10:19587. doi: 10.1038/s41598-020-76350-5 (PMC7658202; doi:10.1038/s41598-020-76350-5)

## Slide 1
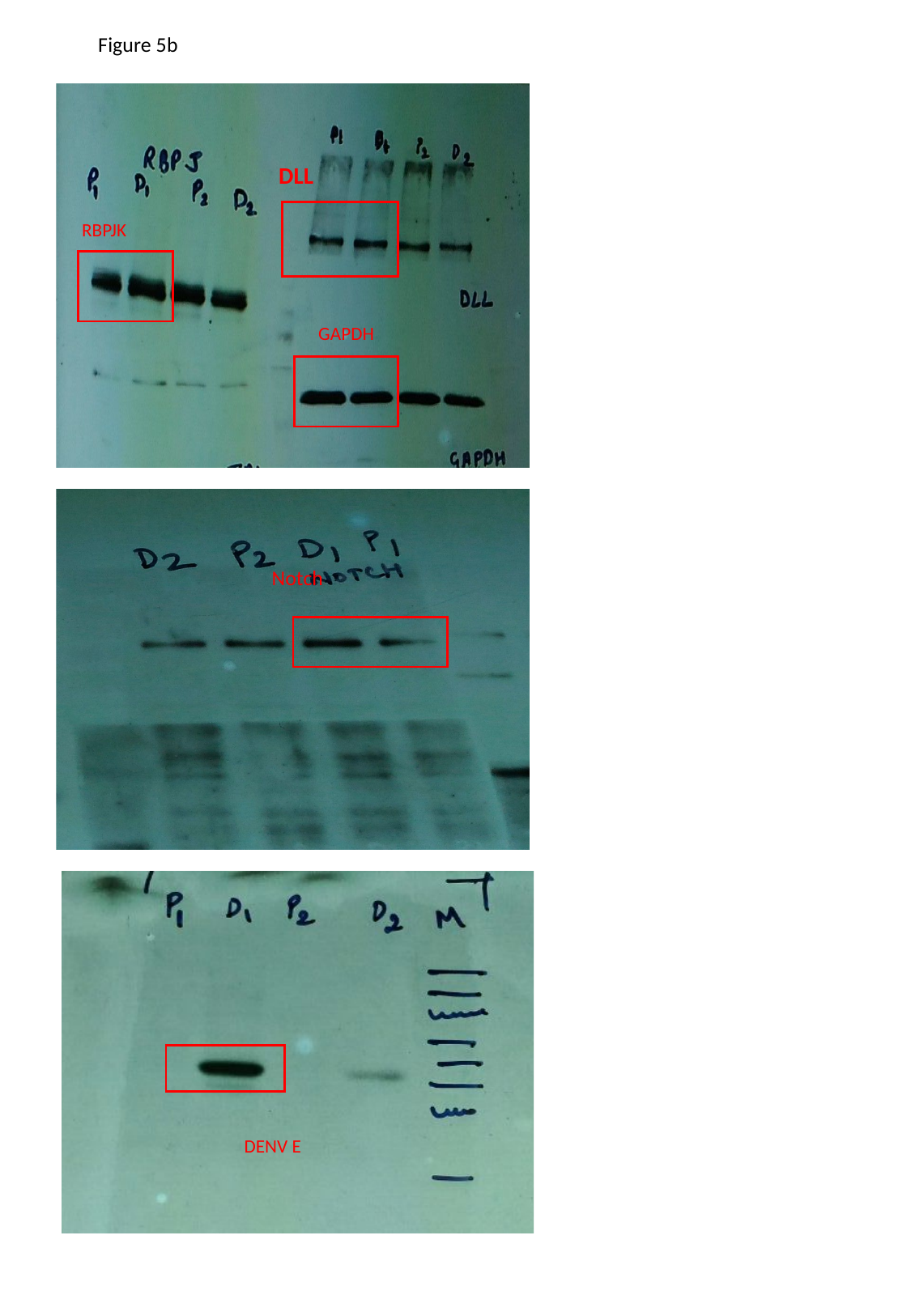

Figure 5b
RBPJK
GAPDH
DLL
Notch
DENV E

## Slide 2
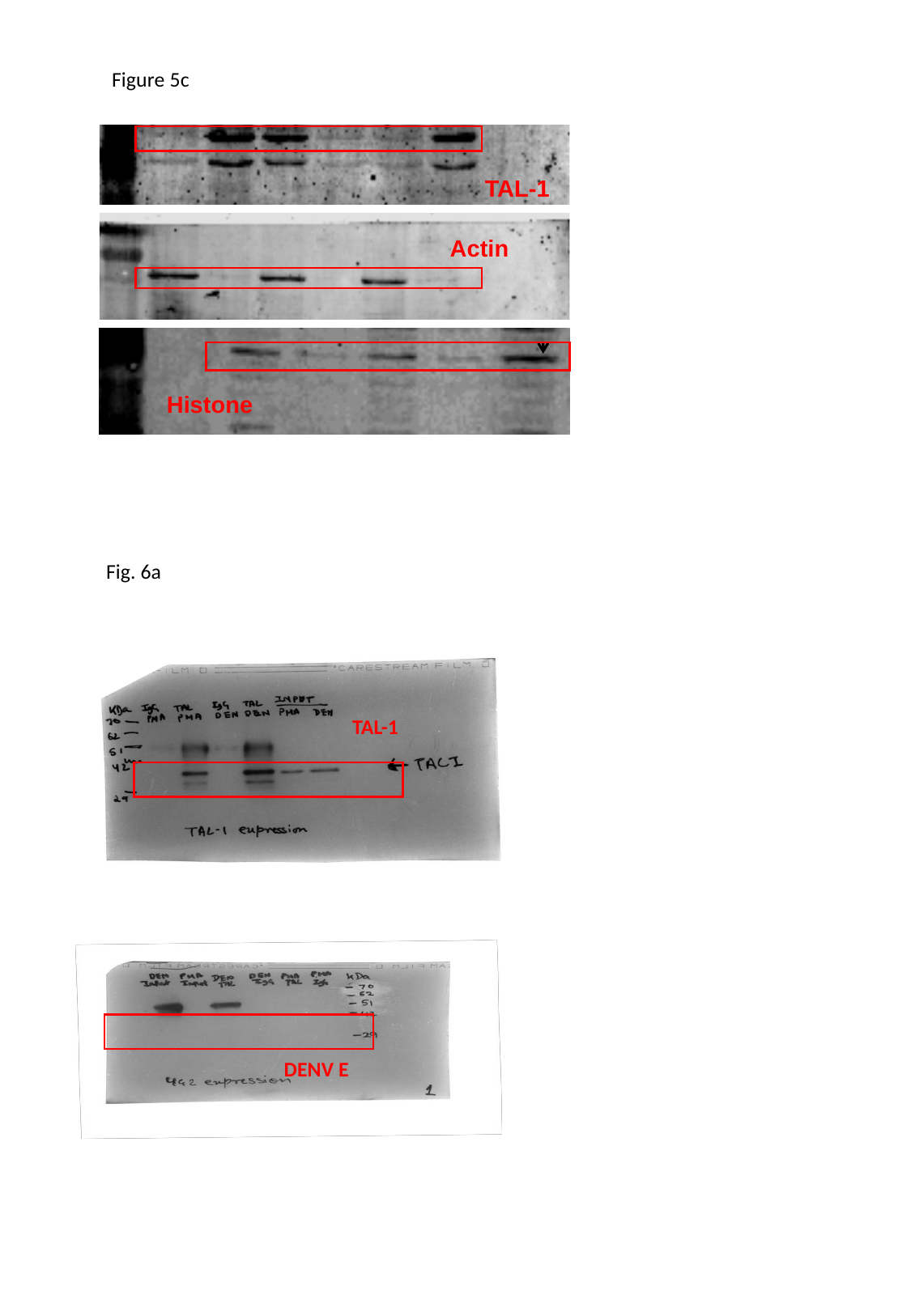

Figure 5c
TAL-1
Actin
Histone
Fig. 6a
TAL-1
DENV E

Supplement: Supplementary file 1 — Supplementary Information. [file 41598_2020_76350_MOESM1_ESM.pptx]
